# Maxwellian Distribution-Based Hall Transport Coefficients for Charged Particles in Magnetic Disk Array

**DOI:** 10.3390/e27030244

**Published:** 2025-02-26

**Authors:** Linlin An, Peifeng Fan

**Affiliations:** 1 School of Physics, Hefei University of Technology, Hefei 230009, China; 2 School of Physics and Optoelectronic Engineering, Anhui University, Hefei 230601, China

**Keywords:** magnetic disk array, Hall transport, Maxwellian distribution

## Abstract

This study explores Hall transport phenomena by expanding upon prior research on magnetic disk arrays (MDAs). We examine the dynamics of charged particles using collision models akin to those in Lorentzian plasma. Previously, we derived transport coefficients under isotropic and mono-kinetic conditions. In this study, we adopt an anisotropic framework, enhanced by Fourier transformation, and employ the local Maxwellian distribution function. These assumptions allow us to calculate the Hall diffusivity, electrical conductivity, and thermal Hall conductivity tensors. Our findings contribute to a deeper understanding of the Hall transport in magnetic disk arrays and chiral active systems.

## 1. Introduction

Hall transport phenomena, commonly known as odd transport [1], have attracted significant interest in the field of physics in recent times. This interest is driven by the exploration of various Hall effects, such as Hall diffusion [2,3], Hall mobility [4], Hall viscosity [1,5,6,7,8,9,10,11], and thermal Hall effects [12,13,14,15,16]. These phenomena are particularly prevalent in chiral active matter [17], systems that are characterized by a continuous injection of microscopic energy [18], resulting in behaviors that elude traditional equilibrium thermodynamics. Such systems typically consist of particles propelled by internal forces, as observed in active colloids [19] and biological cells [20,21,22], where they exhibit intrinsic chiral motion. The concept of chirality in these systems is intrinsically linked to their breaking of time-reversal or parity symmetry [9,23,24], which leads to unique transport behaviors.

A common method for breaking time-reversal symmetry is by applying a magnetic field. In our previous study [25], we introduced a discrete magnetic field configuration called a magnetic disk array (MDA) to theoretically investigate the dynamics of the charged particles within this setup. The presence of the magnetic field inherently breaks time-reversal symmetry, thus facilitating the emergence of Hall transport phenomena. Our investigation focused on deriving the collision integral and the corresponding Boltzmann equation, subsequently utilizing the assumptions of isotropy and mono-kinetic equilibrium to determine the transport coefficients.

In this study, we advance our theoretical approach by relaxing the assumption of isotropy in the calculation of the perturbation distribution functions. Instead, we employ an anisotropic framework, enhanced by Fourier transformation techniques. Departing from the mono-kinetic distribution function, we adopt the local Maxwellian distribution function, which is more prevalent in physical systems, to characterize the equilibrium state. Under these revised conditions, we calculate the Hall diffusivity tensor, the Hall conductivity tensor, and the thermal Hall conductivity tensor. Moreover, we verify the Einstein relation connecting the diffusivity and conductivity tensors, thereby expanding our understanding of the Hall transport phenomena in chiral active systems. We aim to provide a more comprehensive understanding of the charged particle dynamics in magnetic disk arrays, contributing to advancements in the field of Hall transport phenomena.

The rest of this paper is organized as follows: In Section 2, the linear perturbed distribution function is derived under anisotropic conditions. In Section 3, the Hall diffusivity tensor, the Hall conductivity tensor, and the thermal Hall conductivity tensor are calculated using the Maxwellian equilibrium distribution function.

## 2. Linear Solution to the Boltzmann Equation Under Anisotropic Conditions

Consider a system where charged particles of the α-species are confined to a 2D xoy plane. The distribution function for these particles is represented by $f_{\alpha }\left(t,r,v\right)$, where $r=xe_{x}+ye_{y}$ and $v=v_{x}e_{x}+v_{y}e_{y}$ denote the particle’s position and velocity, respectively. For convenience, we can use complex variables to describe a 2D object, rewriting the position as r=x+iy and the velocity as $v=v_{x}+iv_{y}$, thus associating r with *r* and v with *v*.

In this study, we examine a 2D transport process for the charged particles within a discrete magnetic field configuration characterized by a repeating pattern. The fundamental repeating unit is a localized magnetic field confined to a circular region, oriented in the *z*-direction, which we refer to as a magnetic disk (MD). These magnetic disks (MDs) are arranged on a 2D plane without overlapping, thereby constructing a magnetic disk array (MDA). As charged particles move through this MDA, they will be scattered, and we aim to investigate the transport characteristics within this particular setup. We assume that the motion of the MDs is independent of the scattering process; specifically, if the MDs are initially fixed, they remain stationary and are unaffected by the charged particles. Under these conditions, the system can be described using the Lorentz model [26,27,28], where the MDs are analogous to fixed ions, and the interactions between the charged particles and the MDs are simplified as collisions.

Using the results from Ref. [25], the collision integral in this scenario is expressed as

(1) $${\left(\frac{\partial f_{\alpha }}{\partial t}\right)}_{c}^{\pm }=n_{D}\left(t,r\right)\left|v\right|\left|r_{D}\right|\int_{0}^{\pi }\left[f_{\alpha }\left(t,r,v^{\pm }\right)-f_{\alpha }\left(t,r,v\right)\right]\sin\varphi d\varphi .$$

where $n_{D}\left(t,r\right)$ represents the density of the MDs in the local 2D domain, and $|r_{D}|$ is the radius of an individual MD. Here,

(2) $$v^{\pm }\equiv \frac{\gamma_{\alpha }\mp e^{i\varphi }}{\gamma_{\alpha }\mp e^{-i\varphi }}v$$

denotes the velocity (expressed as a complex variable) at which the particle enters the MD and exits with the velocity *v*. In Equation (2), the parameter $\gamma_{\alpha }$ is the ratio of the gyroradius $|r_{c\alpha }|$ of the charged particle to the radius of the MD, i.e.,

(3) $$\gamma_{\alpha }=\frac{\left|r_{c\alpha }\right|}{\left|r_{D}\right|},$$

where

(4) $$\left|r_{c\alpha }\right|=\frac{m_{\alpha }\left|v\right|c}{q_{\alpha }B_{z}}=\frac{\left|v\right|}{\left|\omega_{c\alpha }\right|},$$

and we have

(5) $$\omega_{c\alpha }=-\frac{q_{\alpha }B_{z}}{m_{\alpha }c}$$

as the gyrofrequency, $B_{z}$ is the magnetic field within MD, *c* is the speed of light, and $q_{\alpha }$ and $m_{\alpha }$ are the charge and mass of the charged particle of the α-species, respectively.

From Equations (1) and (2), we can see how the interactions between charged particles and magnetic disks (MDs) influence the collision integral. It is evident that the collision integral is proportional to the density of the MDs, $n_{D}\left(t,r\right)$, and the radius of the MDs, $r_{D}$. Based on a physical interpretation of the collision integral, this can be understood: the greater the density of the MDs and the larger their radius, the higher the probability that a charged particle will be scattered by the MDA. The contributions of the magnetic field’s strength and particle velocity are implicit in the fundamental ratio $\gamma_{\alpha }$, which is contained within $v^{\pm }$. By rewriting the fundamental ratio as $\gamma_{\alpha }=m_{\alpha }\left|v\right|c/q_{\alpha }B_{z}$, we can observe that both the magnetic field and the particle velocity are factors in the collision integral. Detailed derivations of Equation (1) were presented in our previous work; for more information, see Ref. [25].

In Equations (1) and (2), the superscript “+” corresponds to the scenario where $\omega_{\alpha }>0$, whereas “−” corresponds to the case where $\omega_{\alpha }<0$. Having provided the collision integral, the Boltzmann equation for charged particles is subsequently expressed as follows:

(6) $$\frac{\partial f_{\alpha }}{\partial t}+v\cdot \frac{\partial f_{\alpha }}{\partial r}+\frac{F_{\alpha }}{m_{\alpha }}\cdot \frac{\partial f_{\alpha }}{\partial v}={\left(\frac{\partial f_{\alpha }}{\partial t}\right)}_{c}^{\pm }.$$

We proceed to derive the linear Boltzmann equation and compute the perturbed distribution function. Suppose the distribution function can be decomposed into

(7) $$f_{\alpha }=f_{\alpha 0}+\delta f_{\alpha }^{\pm }$$

where $f_{\alpha 0}$ denotes the local equilibrium distribution, and $\delta f_{\alpha }\ll f_{\alpha 0}$ represents a small deviation from $f_{\alpha 0}$. Substituting Equation (7) into Equation (6) and neglecting the higher-order terms, we obtain the linear Boltzmann equation

(8) $$\begin{array}{c} \frac{\partial \delta f_{\alpha }^{\pm }}{\partial t}+v\cdot \nabla f_{0\alpha }+\frac{F_{\alpha }}{m_{\alpha }}\cdot \frac{\partial f_{0\alpha }}{\partial v}\\ =n_{D}\left(t,r\right)\left|v\right|\left|r_{D}\right|\int_{0}^{\pi }\left[\delta f_{\alpha }^{\pm }\left(t,r,v^{\pm }\right)-\delta f_{\alpha }^{\pm }\left(t,r,v\right)\right]\sin\varphi d\varphi -U^{\pm }\left(f_{\alpha 0}\right), \end{array}$$

where

(9) $$U^{\pm }\left(f_{\alpha 0}\right)=-n_{D}\left(t,r\right)\left|v\right|\left|r_{D}\right|\int_{0}^{\pi }\left[f_{\alpha 0}\left(t,r,v^{\pm }\right)-f_{\alpha 0}\left(t,r,v\right)\right]\sin\varphi d\varphi .$$

Since $\left|v^{\pm }\right|=\left|v\right|$, we can express *v* and $v^{\pm }$ as

(10) $$\begin{array}{c} v=\left|v\right|e^{i\xi },\;v^{\pm }=\left|v\right|e^{i\xi^{\pm }}, \end{array}$$

(11) $$\begin{array}{c} e^{i\xi^{\pm }}=\frac{\gamma_{\alpha }-s_{\alpha }e^{i\varphi }}{\gamma_{\alpha }-s_{\alpha }e^{-i\varphi }}e^{i\xi }. \end{array}$$

Since $\delta f_{\alpha }\left(t,r,v\right)$ can be considered a function of |v| and ξ, and is periodic with respect to ξ, we can solve Equation (8) using Fourier’s method. Assuming that $\partial \delta f_{\alpha }^{\pm }/\partial t=-i\omega \delta f_{\alpha }^{\pm }$ and considering that $\delta f_{\alpha m}^{\pm }=\delta f_{\alpha m}^{\pm }\left(t,r,\left|v\right|\right)$ and $U_{m}^{\pm }=U_{m}^{\pm }\left(t,r,\left|v\right|\right)$ are the Fourier components of $\delta f_{\alpha }^{\pm }$ and $U^{\pm }$, respectively, this means that $\delta f_{\alpha }^{\pm }$ and $U^{\pm }$ can be expressed as

(12) $$\begin{array}{c} \delta f_{\alpha }^{\pm }=\sum_{m=-\infty }^{+\infty }\delta f_{\alpha m}^{\pm }e^{im\xi },\;\delta f_{\alpha m}^{\pm }=\frac{1}{2\pi }\int_{0}^{2\pi }\delta f_{\alpha }^{\pm }e^{-im\xi }d\xi , \end{array}$$

(13) $$\begin{array}{c} U^{\pm }=\sum_{m=-\infty }^{+\infty }U_{m}^{\pm }e^{im\xi },\;U_{m}^{\pm }=\frac{1}{2\pi }\int_{0}^{2\pi }U^{\pm }e^{-im\xi }d\xi . \end{array}$$

Substituting Equations (12) and (13) into Equation (8) can be rewritten as

(14) $$-i\omega \sum_{m=-\infty }^{+\infty }\delta f_{\alpha m}^{\pm }e^{im\xi }+v\cdot \nabla f_{0\alpha }+\frac{F_{\alpha }}{m_{\alpha }}\cdot \frac{\partial f_{0\alpha }}{\partial v}=-\sum_{m=-\infty }^{+\infty }\nu_{\alpha m}^{\pm }\delta f_{\alpha m}^{\pm }e^{im\xi }-\sum_{m=-\infty }^{+\infty }U_{m}^{\pm }e^{im\xi },$$

using Equations (10) and (11), where $F_{\alpha }$ denotes the external force. Here, $\nu_{\alpha m}^{\pm }$ is defined as

(15) $$\nu_{\alpha m}^{\pm }=\nu_{\alpha m}^{\pm }\left(t,r,\left|v\right|\right)\equiv n_{D}\left(t,r\right)\left|v\right|\left|r_{D}\right|K_{m}^{\pm }\left(\gamma_{\alpha }\right),$$

where $K_{m}^{\pm }\left(\gamma_{\alpha }\right)$ is a special integral function given by

(16) $$K_{m}^{\pm }\left(\gamma_{\alpha }\right)\equiv \int_{0}^{\pi }\left[1-{\left(\frac{\gamma_{\alpha }\mp e^{i\varphi }}{\gamma_{\alpha }\mp e^{-i\varphi }}\right)}^{m}\right]\sin\varphi d\varphi$$

This function (Appendix A) satisfies the following basic properties

(17) $$\begin{array}{c} K_{0}^{\pm }\left(\gamma_{\alpha }\right)\equiv 0, \end{array}$$

(18) $$\begin{array}{c} {\left[K_{m}^{\pm }\left(\gamma_{\alpha }\right)\right]}^{*}=K_{-m}^{\pm }\left(\gamma_{\alpha }\right), \end{array}$$

(19) $$\begin{array}{c} {\left[K_{m}^{+}\left(\gamma_{\alpha }\right)\right]}^{*}=K_{m}^{-}\left(\gamma_{\alpha }\right), \end{array}$$

(20) $$\begin{array}{c} {\left[K_{m}^{\pm }\left(-\gamma_{\alpha }\right)\right]}^{*}=K_{m}^{\pm }\left(\gamma_{\alpha }\right). \end{array}$$

By reorganizing the terms in Equation (14), we arrive at the following expression:

(21) $$v\cdot \nabla f_{0\alpha }+\frac{F_{\alpha }}{m_{\alpha }}\cdot \frac{\partial f_{0\alpha }}{\partial v}=-\sum_{m=-\infty }^{+\infty }\left[\left(\nu_{\alpha m}^{\pm }-i\omega \right)\delta f_{\alpha m}^{\pm }+U_{m}^{\pm }\right]e^{im\xi }.$$

The Fourier coefficients on the left-hand side (LHS) of Equation (21) are given by $\left(e^{im\xi },v\cdot \nabla f_{0\alpha }\right.$ Fα/mα+Fα/mα·∂f0α/∂v/2π. Consequently, Equation (21) can be simplified into

(22) $$\left(\nu_{\alpha m}^{\pm }-i\omega \right)\delta f_{\alpha m}^{\pm }=-\frac{1}{2\pi }\left(e^{im\xi },v\cdot \nabla f_{0\alpha }+\frac{F_{\alpha }}{m_{\alpha }}\cdot \frac{\partial f_{0\alpha }}{\partial v}\right)-U_{m}^{\pm },$$

Here, the notation $\left(\;,\;\right)$ denotes the inner product of any two complex functions $h\left(\xi \right)$ and $g\left(\xi \right)$, defined as

(23) $$\left(h\left(\xi \right),g\left(\xi \right)\right):=\int_{0}^{2\pi }h^{*}\left(\xi \right)g\left(\xi \right)d\xi .$$

Next, we determine the perturbed distribution function $\delta f_{\alpha }^{\pm }$ using Equation (22). For a case where m=0, we find that $\nu_{\alpha 0}^{\pm }=0$ using Equations (15) and (17). Equation (22) then is simplified into

(24) $$i\omega \delta f_{\alpha 0}^{\pm }=\frac{1}{2\pi }\int_{0}^{2\pi }\left(v\cdot \nabla f_{0\alpha }+\frac{F_{\alpha }}{m_{\alpha }}\cdot \frac{\partial f_{0\alpha }}{\partial v}\right)d\xi +U_{0}^{\pm }.$$

If the right-hand side (RHS) of Equation (24) is not zero, we cannot set the frequency ω to zero to nullify the LHS of Equation (24). Consequently, it is not possible to achieve a time-independent or stable distribution function, meaning that a dynamic equilibrium transport scenario cannot exist. However, if the equilibrium distribution $f_{\alpha 0}$ satisfies the isotropic condition—meaning $f_{0\alpha }$ is independent of ξ—then we have $f_{\alpha 0}\left(t,r,v^{\pm }\right)=f_{\alpha 0}\left(t,r,v\right)$, resulting in $U^{\pm }\left(f_{\alpha 0}\right)=0$ and $U_{0}^{\pm }=0$. The first term on the RHS of Equation (24) can also be shown to be zero (see Ref. [25]). In this situation, the frequency ω can be set to zero, allowing dynamic equilibrium to exist.

For m≠0, we have

(25) $$\delta f_{\alpha m}^{\pm }=-\frac{1}{2\pi \left(\nu_{\alpha m}^{\pm }-i\omega \right)}\left(e^{im\xi },v\cdot \nabla f_{0\alpha }+\frac{F_{\alpha }}{m_{\alpha }}\cdot \frac{\partial f_{0\alpha }}{\partial v}\right)-\frac{U_{m}^{\pm }}{\nu_{\alpha m}^{\pm }-i\omega }$$

Combining Equations (24) and (25), we obtain the perturbed distribution function

(26) $$\delta f_{\alpha }^{\pm }=-\sum_{m=-\infty }^{+\infty }\left[\frac{1}{2\pi \left(\nu_{\alpha m}^{\pm }-i\omega \right)}\left(e^{im\xi },v\cdot \nabla f_{0\alpha }+\frac{F_{\alpha }}{m_{\alpha }}\cdot \frac{\partial f_{0\alpha }}{\partial v}\right)+\frac{U_{m}^{\pm }}{\nu_{\alpha m}^{\pm }-i\omega }\right]e^{im\xi }.$$

## 3. Hall Transport Coefficients with the Local Maxwellian Equilibrium Distribution

We now assume that the local equilibrium distribution $f_{\alpha 0}$ is the 2D local Maxwellian distribution function, given by

(27) $$\begin{array}{c} f_{\alpha 0}\left(r,v\right)=f_{\alpha M}\left(r,v\right)\equiv \frac{m_{\alpha }n_{\alpha }\left(r\right)}{2\pi T_{\alpha }\left(r\right)}\exp\left[-\frac{m_{\alpha }v^{2}}{2T_{\alpha }\left(r\right)}\right], \end{array}$$

where $n_{\alpha }\left(r\right)$ and $T_{\alpha }\left(r\right)$ are the local density and temperature of the charged particles of the α-species. Here, we have set the Boltzmann constant $k_{B}=1$.

Under these conditions, the isotropy condition is met, with $U_{m}^{\pm }=0$. As discussed in Section 2, dynamic equilibrium can be established. This stable transport scenario (ω=0) can be utilized to determine the transport coefficients. The key inner product is simplified into

(28) $$\left(e^{im\xi },v\cdot \nabla f_{\alpha 0}+\frac{F_{\alpha }}{m_{\alpha }}\cdot \frac{\partial f_{0\alpha }}{\partial v}\right)=\left({\hat{D}}_{\alpha }f_{\alpha 0}\right)\cdot \left(e^{im\xi },e_{v}\right),$$

where $e_{v}=v/\left|v\right|$ is the unit vector in the radial direction of velocity space, satisfying

(29) $$e_{v}=\cos\xi e_{x}+\sin\xi e_{y},$$

and the operator ${\hat{D}}_{\alpha }$ is defined as

(30) $${\hat{D}}_{\alpha }\equiv \left|v\right|\frac{\partial }{\partial r}+\frac{F_{\alpha }}{m_{\alpha }}\frac{\partial }{\partial \left|v\right|}.$$

The last term on the right-hand side of Equation (28) can be directly integrated as

(31) $$\left(e^{im\xi },e_{v}\right)=\left\{\begin{array}{cc} \pi \left(e_{x}-ie_{y}\right), & m=1,\\ \pi \left(e_{x}+ie_{y}\right), & m=-1,\\ 0, & m\neq \pm 1. \end{array}\right.$$

Substituting Equations (28), (30), and (31) into Equation (2), the perturbed distribution function is simplified into

(32) $$\delta f_{\alpha }^{\pm }\left(r,v\right)=-e_{v}\cdot M_{\alpha }^{\pm }\left(r,\left|v\right|\right)\cdot {\hat{D}}_{\alpha }f_{\alpha 0},$$

In Equation (32), the tensor $M_{\alpha }^{\pm }$ is characterized by the matrix representation

(33) $$M_{\alpha }^{\pm }\left(r,\left|v\right|\right)=\left(\begin{array}{cc} \tau_{\alpha 1} & \mp \tau_{\alpha 2}\\ \pm \tau_{\alpha 2} & \tau_{\alpha 1} \end{array}\right),$$

where $\tau_{\alpha 1}$ and $\tau_{\alpha 2}$ are related to the inverse of $\nu_{\alpha 1}^{\pm }\left(r,\left|v\right|\right)$ (see Equation (15)), defined as

(34) $$\frac{1}{\nu_{\alpha 1}^{\pm }\left(r,\left|v\right|\right)}\tau_{\alpha 1}\left(r,\left|v\right|\right)\mp i\;\tau_{\alpha 2}\left(r,\left|v\right|\right).$$

It can easily be seen that the off-diagonal elements of $M_{\alpha }^{\pm }\left(r,\left|v\right|\right)$ in Equation (33) are antisymmetric, i.e.,

(35) $$M_{\alpha ij}^{\pm }=-M_{\alpha ji}^{\pm },\;i\neq j.$$

We next utilize the fundamental equations outlined above to determine the transport coefficients.

### 3.1. The Hall Diffusivity Tensor

We begin by calculating the diffusion tensor. Assuming that the external force $F_{\alpha }=0$ and the temperature are uniform throughout the entire space such that $T_{\alpha }\left(r\right)=T_{\alpha 0}$ is a constant, the operator ${\hat{D}}_{\alpha }$ is then reduced to ${\hat{D}}_{\alpha }=\left|v\right|\partial /\partial r$. Consequently, the perturbed distribution function $\delta f_{\alpha }^{\pm }$ can then be expressed as

(36) $$\delta f_{\alpha }^{\pm }\left(t,r,v\right)=-e_{v}\cdot M_{\alpha }^{\pm }\left(r,\left|v\right|\right)\cdot \left|v\right|\frac{\partial f_{\alpha 0}\left(r,v\right)}{\partial r}.$$

Substituting Equation (27) into Equation (36), $\delta f_{\alpha }^{\pm }\left(t,r,v\right)$ can be transformed into

(37) $$\delta f_{\alpha }^{\pm }\left(t,r,v\right)=-\frac{\left|v\right|f_{\alpha M}\left(r,v\right)}{n_{\alpha }\left(r\right)}e_{v}\cdot M_{\alpha }^{\pm }\left(r,\left|v\right|\right)\cdot \nabla n_{\alpha }\left(r\right).$$

Using Equation (37), the particle flux of the α-species can be calculated as

(38) $$\begin{array}{cc} \Gamma_{\alpha }^{\pm }\left(r\right) & =\int f_{\alpha }\left(t,r,v\right)vd^{2}v=\int \left[f_{\alpha M}\left(r,v\right)+\delta f_{\alpha }^{\pm }\left(t,r,v\right)\right]vd^{2}v\;\\ \; & =\int \delta f_{\alpha }^{\pm }\left(t,r,v\right)vd^{2}v\;\\ \; & =-\frac{1}{n_{\alpha }\left(r\right)}\int \left|v\right|f_{\alpha M}\left(r,v\right)e_{v}\cdot M_{\alpha }^{\pm }\left(r,\left|v\right|\right)\cdot \nabla n_{\alpha }\left(r\right)\left|v\right|e_{v}d^{2}v\;\\ \; & =-\frac{1}{n_{\alpha }\left(r\right)}\left\{\left[\int_{0}^{2\pi }e_{v}e_{v}d\xi \right]\cdot \left[\int_{0}^{+\infty }f_{\alpha M}\left(r,v\right)M_{\alpha }^{\pm }\left(r,\left|v\right|\right){\left|v\right|}^{3}d\left|v\right|\right]\right\}\cdot \nabla n_{\alpha }\left(r\right)\;\\ \; & =-D_{\alpha }^{\pm }\left(r\right)\cdot \nabla n_{\alpha }\left(r\right), \end{array}$$

where

(39) $$D_{\alpha }^{\pm }\left(r\right)=\frac{\pi }{n_{\alpha }\left(r\right)}\int_{0}^{+\infty }f_{\alpha M}\left(r,v\right)M_{\alpha }^{\pm }\left(r,\left|v\right|\right){\left|v\right|}^{3}dv$$

is the Hall diffusivity tensor. In deriving Equation (38), we use

(40) $$\begin{array}{cc} \; & \int_{0}^{2\pi }e_{v}e_{v}d\xi =\int_{0}^{2\pi }\left(\cos\xi e_{x}+\sin\xi e_{y}\right)\left(\cos\xi e_{x}+\sin\xi e_{y}\right)d\xi \;\\ \; & =\int_{0}^{2\pi }\left[\cos^{2}\xi e_{x}e_{x}+\cos\xi \sin\xi \left(e_{x}e_{y}+e_{y}e_{x}\right)+\sin^{2}\xi e_{y}e_{y}\right]d\xi \;\\ \; & =\pi \left(e_{x}e_{x}+e_{y}e_{y}\right)=\pi I, \end{array}$$

where I is the 2D identity tensor. Since the off-diagonal elements of $M_{\alpha }^{\pm }\left(r,\left|v\right|\right)$ in Equation (39) are antisymmetric, this renders the Hall diffusivity tensor “odd”. Then the off-diagonal elements of $D_{\alpha }^{\pm }\left(r\right)$ are antisymmetric and can be expressed as

(41) $$D_{\alpha }^{\pm }\left(r\right)=\left(\begin{array}{cc} A_{\alpha } & \mp B_{\alpha }\\ \pm B_{\alpha } & A_{\alpha } \end{array}\right),$$

where $A_{\alpha },B_{\alpha }>0$ is a function of r, which can be determined from Equation (39).

For the density gradient along the *x*-axis, $\nabla n=\left(\partial_{x}n\right)e_{x}$, the particle flux becomes

(42) $$\Gamma_{\alpha }^{\pm }\left(r\right)=-\partial_{x}n_{\alpha }\left(A_{\alpha }e_{x}\pm B_{\alpha }e_{y}\right).$$

Equation (42) indicates that even if the particle density varies only along the *x*-axis, the flux can still have a *y*-direction component. This phenomenon can be understood according to the micromotion of the particles scattered by the magnetic field.

For example, the electrons scattered by a magnetic field in the *z*-direction move according to an anticlockwise trajectory. The projection of the flux along the *y*-axis becomes

(43) $$e_{y}\cdot \Gamma_{\alpha }^{+}\left(r\right)=-\left(\partial_{x}n_{\alpha }\right)B_{\alpha }>0,$$

when $\partial_{x}n_{\alpha }<0$. On the other hand, for the ions scattered by a *z*-direction magnetic field, the electrons move clockwise, and the projection of flux along the *y*-axis becomes

(44) $$e_{y}\cdot \Gamma_{\alpha }^{-}\left(r\right)=-\left(\partial_{x}n_{\alpha }\right)B_{\alpha }<0,$$

when $\partial_{x}n_{\alpha }<0$.

Using the continuity equation $\partial_{t}n_{\alpha }+\nabla \cdot \Gamma_{\alpha }^{\pm }\left(r\right)=0$, we can derive the diffusion equation as

(45) $$\partial_{t}n_{\alpha }=\nabla \cdot \left[D_{\alpha }^{\pm }\left(r\right)\cdot \nabla n_{\alpha }\right].$$

**Figure 1:**
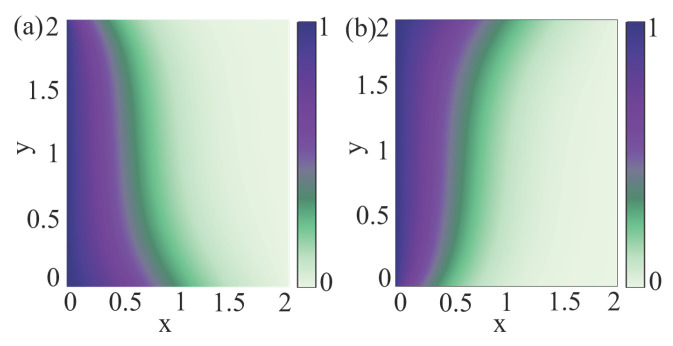
Particle density profile after time evolution: (**a**) particle density corresponding to the diffusion tensor ${\tilde{D}}_{\alpha }^{-}$ at $\tilde{t}=0.2$; (**b**) particle density corresponding to the diffusion tensor ${\tilde{D}}_{\alpha }^{+}$ at $\tilde{t}=0.2$.

Equation (45) can be non-dimensionalized as follows:

(46) $$\partial_{\tilde{t}}{\tilde{n}}_{\alpha }=\tilde{\nabla }\cdot \left[{\tilde{D}}_{\alpha }^{\pm }\left(r\right)\cdot \tilde{\nabla }{\tilde{n}}_{\alpha }\right].$$

where $\tilde{t}=\left|\omega_{c\alpha }\right|t$, $\tilde{r}=r/\left|r_{D}\right|$, ${\tilde{n}}_{\alpha }=\pi {\left|r_{D}\right|}^{2}n_{\alpha }$, and ${\tilde{D}}_{\alpha }^{\pm }=D_{\alpha }^{\pm }/\left|\omega_{c\alpha }\right|{\left|r_{D}\right|}^{2}$ are the corresponding non-dimensional quantities. In Figure 1, we present a simulation of the particle diffusion process. We assume that $D_{\alpha }^{\pm }\left(r\right)$ is a constant tensor, leading to $A_{\alpha }$ and $B_{\alpha }$ being constants. We set the parameters to $A_{\alpha }=1$ and $B_{\alpha }=\pi /4$. The boundary conditions are defined as follows: a flux condition is imposed at the top and bottom boundaries, ensuring a zero net particle flux across these boundaries, $\Gamma_{\alpha }^{\pm }=0$. Dirichlet boundary conditions are applied at the left and right boundaries to specify the particle density. At the left boundary, the density is fixed at $\tilde{n}\left(\tilde{t},\tilde{x}=0,\tilde{y}\right)={\tilde{n}}_{0}$, where ${\tilde{n}}_{0}=1$. At the right boundary, the density is set to $\tilde{n}\left(\tilde{t},\tilde{x}=2,\tilde{y}\right)=0$. The initial particle density follows a Gaussian distribution, $\tilde{n}\left(\tilde{t}=0,\tilde{x},\tilde{y}\right)={\tilde{n}}_{0}e^{-{\tilde{x}}^{2}/L^{2}}$, with L=0.08. Form Figure 1, we observe that the particles move “downward” for the $D_{\alpha }^{-}$ tensor and “upward” for the $D_{\alpha }^{+}$ tensor, consistent with Equations (43) and (44), respectively.

### 3.2. The Hall Conductivity Tensor

To derive the Hall conductivity tensor, we assume that the system is under a uniform electric field $E=E_{x}e_{x}+E_{y}e_{y}$ and the force field acting on the α-species is thus given by $F_{\alpha }=q_{\alpha }E.$ Assume the system is composed of electrons and ions, with the ions stationary on the timescale of electron motion since electrons move much faster due to their lighter mass. The electron density and temperature are uniform throughout the system, meaning $n_{e}\left(r\right)=n_{e0}$ and $T_{e}\left(r\right)=T_{e0}$ are constants. In this scenario, the operator becomes ${\hat{D}}_{\alpha }=\left(q_{\alpha }E/m_{\alpha }\right)\partial /\partial \left|v\right|$. Consequently, the perturbed distribution functions $\delta f_{e}^{\pm }$ and $\delta f_{i}^{\pm }$ become

(47) $$\begin{array}{cc} \delta f_{e}^{\pm }\left(t,r,v\right) & =-\frac{e|v|f_{eM}\left(r,v\right)}{T_{e0}}e_{v}\cdot M_{e}^{\pm }\left(r,\left|v\right|\right)\cdot E, \end{array}$$

(48) $$\begin{array}{cc} \delta f_{i}^{\pm }\left(t,r,v\right) & =0. \end{array}$$

The electric current, defined as $J^{\pm }=\sum_{\alpha }q_{\alpha }\int f_{\alpha }\left(t,r,v\right)vd^{2}v$, is then calculated as

(49) $$\begin{array}{cc} J^{\pm } & =-e\int \left(f_{eM}\left(r,v\right)+\delta f_{e}^{\pm }\left(t,r,v\right)\right)vd^{2}v\;\\ \; & =-e\int \delta f_{e}^{\pm }\left(t,r,v\right)vd^{2}v=\frac{e^{2}}{T_{e0}}\int \left[\left|v\right|f_{eM}\left(r,v\right)e_{v}\cdot M_{e}^{\pm }\left(r,\left|v\right|\right)\cdot E\right]vd^{2}v\;\\ \; & =\frac{e^{2}}{T_{\alpha 0}}\left[\int_{0}^{2\pi }e_{v}e_{v}d\xi \right]\cdot \left[\int_{0}^{+\infty }f_{eM}\left(r,v\right)M_{e}^{\pm }\left(r,\left|v\right|\right){\left|v\right|}^{3}d\left|v\right|\right]\cdot E\;\\ \; & =\sigma_{e}^{\pm }\cdot E, \end{array}$$

where $\sigma_{e}^{\pm }$ is the Hall conductivity tensor, defined by

(50) $$\sigma_{e}^{\pm }=\frac{\pi e^{2}}{T_{e0}}\int_{0}^{+\infty }f_{eM}\left(r,v\right)M_{e}^{\pm }\left(r,\left|v\right|\right){\left|v\right|}^{3}d\left|v\right|.$$

By comparing Equations (39) and (50), we find that

(51) $$D_{e}^{\pm }=\frac{T_{e0}}{e^{2}n_{e0}}\sigma_{e}^{\pm }$$

which is consistent with the Einstein relation.

### 3.3. The Thermal Hall Conductivity Tensor

Finally, we analyze the heat transport and calculate the thermal Hall conductivity tensor. We consider a system comprising two species of particles: electrons and ions. In this context, the ions are cold and relatively stationary compared to the hot, mobile electrons. Assuming there is no equilibrium flow and no external force field, we have $F_{\alpha }=0.$ The temperature is now position-dependent, r, creating a temperature gradient ($\nabla T_{e}\neq 0$). The electron pressure follows the ideal gas equation of state

(52) $$P_{e}\left(r\right)=n_{e}\left(r\right)T_{e}\left(r\right).$$

The condition of no equilibrium flow suggests that the electron pressure is constant across the two-dimensional plane; that is, $P_{e}=P_{e0}$ is a constant or $\nabla P_{e}=0$. By applying the gradient operation to the ideal gas Equation (52), we obtain

(53) $$\frac{\nabla n_{e}\left(r\right)}{n_{e}\left(r\right)}=-\frac{\nabla T_{e}\left(r\right)}{T_{e}\left(r\right)}.$$

This equation implies that the electron density cannot be uniform across the 2D plane. The operator is then reduced into ${\hat{D}}_{e}=\left|v\right|\partial /\partial r$. We substitute Equation (27) into (32) and the perturbed distribution function $\delta f_{e}^{\pm }$ for the electrons can be transformed into

(54) $$\begin{array}{cc} \; & \delta f_{e}^{\pm }\left(t,r,v\right)=-e_{v}\cdot M_{e}^{\pm }\left(r,\left|v\right|\right)\cdot \left|v\right|\frac{\partial f_{eM}\left(r,v\right)}{\partial r}\;\\ \; & =-f_{eM}\left(r,v\right)\left|v\right|e_{v}\cdot M_{e}^{\pm }\left(r,\left|v\right|\right)\cdot \left[\frac{\nabla n_{e}\left(r\right)}{n_{e}\left(r\right)}-\frac{\nabla T_{e}\left(r\right)}{T_{e}\left(r\right)}+\frac{m_{e}v^{2}}{2T_{e}^{2}\left(r\right)}\nabla T_{e}\left(r\right)\right]. \end{array}$$

Substituting Equation (53) into Equation (54), we obtain

(55) $$\delta f_{e}^{\pm }\left(t,r,v\right)=-f_{eM}\left(r,v\right)\left[\frac{m_{e}v^{2}/2-2T_{e}\left(r\right)}{T_{e}^{2}\left(r\right)}\right]\left|v\right|e_{v}\cdot M_{e}^{\pm }\left(r,\left|v\right|\right)\cdot \nabla T_{e}\left(r\right).$$

Using Equation (55), the electron heat flux $q_{e}^{\pm }$ is then determined as

(56) $$\begin{array}{cc} q_{e}^{\pm }=\frac{1}{2}m_{e}\int f_{e}\left(t,r,v\right)v^{2}vd^{2}v\;\\ \; & =\frac{1}{2}m_{e}\int \left[f_{eM}\left(r,v\right)+\delta f_{e}^{\pm }\left(t,r,v\right)\right]v^{2}vd^{2}v=\frac{1}{2}m_{e}\int \delta f_{e}^{\pm }\left(t,r,v\right)v^{2}vd^{2}v\;\\ \; & =-\frac{m_{e}}{2}\left[\int_{0}^{2\pi }e_{v}e_{v}d\xi \right]\cdot \left[\int_{0}^{+\infty }f_{eM}\left(r,v\right)\left[\frac{m{\left|v\right|}^{2}/2-2T_{e}\left(r\right)}{T_{e}^{2}\left(r\right)}\right]{\left|v\right|}^{5}M_{e}^{\pm }\left(r,\left|v\right|\right)d\left|v\right|\right]\cdot \nabla T_{e}\left(r\right)\;\\ \; & =-\kappa_{e}^{\pm }\cdot \nabla T_{e}\left(r\right) \end{array}$$

where $\kappa_{e}^{\pm }$ is the electron thermal Hall conductivity tensor, defined as

(57) $$\kappa_{e}^{\pm }=\frac{\pi m_{e}}{2}\int_{0}^{+\infty }f_{eM}\left(r,v\right)\left[\frac{m_{e}{\left|v\right|}^{2}/2-2T_{e}\left(r\right)}{T_{e}^{2}\left(r\right)}\right]{\left|v\right|}^{5}M_{e}^{\pm }\left(r,\left|v\right|\right)d\left|v\right|.$$

Since the off-diagonal elements of $M_{e}^{\pm }\left(r,\left|v\right|\right)$ in Equation (57) are antisymmetric, this renders the electron thermal Hall conductivity tensor “odd”.

## 4. Conclusions

This study has advanced our understanding of the Hall transport phenomena in magnetic disk arrays (MDAs), where the discrete magnetic field configuration facilitates the emergence of Hall effects by breaking time-reversal symmetry. As a natural progression of this work, we propose two potential future directions. First, the investigation of magnetic dipole arrays in 3D systems could provide a more comprehensive understanding of the role of spatial dimensions in Hall transport. Second, the exploration of dynamic MDAs, where the time-dependent evolution of the magnetic field is controlled by external coils, could reveal how varying magnetic field conditions influence particle transport. These future studies would not only expand the theoretical framework of Hall transport but also potentially inform the development of novel technologies that harness these phenomena.

The introduction of the MDA concept in this work holds promise for influencing a range of related fields. For example, in magnetohydrodynamics, MDAs could be used to study MHD waves in periodic structures, potentially enabling the development of plasma wave filters with band gap characteristics. Furthermore, the structured magnetic fields in MDAs create unique transport behaviors for charged particles. The transport coefficients derived here provide a foundation for understanding these behaviors and could guide future studies aiming to optimize the particle dynamics in such systems.

Finally, in both laboratory and natural plasma environments, the Hall transport tensor remains a critical tool for describing the diffusion and drift of charged particles under the influence of magnetic fields. The results of this study can contribute to refining theoretical models and simulations of magnetized plasmas, broadening their applicability to practical systems and experimental setups.

## Data Availability

The original contributions presented in this study are included in the article. Further inquiries can be directed to the corresponding author.

## Appendix A. Proofs of Equations (18)–(20) for the $K_{m}^{\pm }$ Function

Initially, we define the $K_{m}^{\pm }$ function as a function of the variable *x*. The function $K_{m}^{\pm }\left(x\right)$ can be rewritten as follows:

(A1) $$\begin{array}{cc} K_{m}^{+}\left(x\right) & =\int_{0}^{\pi }\left[1-{\left(\frac{x-e^{i\varphi }}{x-e^{-i\varphi }}\right)}^{m}\right]\sin\varphi d\varphi , \end{array}$$

(A2) $$\begin{array}{cc} K_{m}^{-}\left(x\right) & =\int_{0}^{\pi }\left[1-{\left(\frac{x+e^{i\varphi }}{x+e^{-i\varphi }}\right)}^{m}\right]\sin\varphi d\varphi . \end{array}$$

Based on Equations (A1) and (A2), we provide a detailed proof for Equations (18)–(20).

Firstly, we present the proofs for Equation (18). Using Equations (A1) and (A2), we can directly calculate ${\left[K_{-m}^{\pm }\left(x\right)\right]}^{*}$ as follows:

(A3) $$\begin{array}{cc} \; & {\left[K_{-m}^{\pm }\left(x\right)\right]}^{*}=\int_{0}^{\pi }{\left[1-{\left(\frac{x-\left(\pm \right)e^{i\varphi }}{x-\left(\pm \right)e^{-i\varphi }}\right)}^{-m}\right]}^{*}\sin\varphi d\varphi \\ \; & =\int_{0}^{\pi }{\left[1-{\left(\frac{x-\left(\pm \right)e^{-i\varphi }}{x-\left(\pm \right)e^{i\varphi }}\right)}^{m}\right]}^{*}\sin\varphi d\varphi \\ \; & =\int_{0}^{\pi }\left[1-{\left(\frac{x-\left(\pm \right)e^{i\varphi }}{x-\left(\pm \right)e^{-i\varphi }}\right)}^{m}\right]\sin\varphi d\varphi =K_{m}^{\pm }\left(x\right). \end{array}$$

Next, we provide the detailed proofs for Equation (19). By introducing the new variable $\varphi^{\prime }=-\varphi +\pi$, we can rewrite Equation (A1) as

(A4) $$\begin{array}{cc} \; & K_{m}^{+}\left(x\right)=\int_{0}^{\pi }\left[1-{\left(\frac{x-e^{i\left(-\varphi^{\prime }+\pi \right)}}{x-e^{-i\left(-\varphi^{\prime }+\pi \right)}}\right)}^{m}\right]\sin\left(-\varphi^{\prime }+\pi \right)d\left(-\varphi^{\prime }+\pi \right)\;\\ \; & =-\int_{\pi }^{0}\left[1-{\left(\frac{x+e^{-i\varphi^{\prime }}}{x+e^{i\varphi^{\prime }}}\right)}^{m}\right]\sin\varphi^{\prime }d\varphi^{\prime }=\int_{0}^{\pi }\left[1-{\left(\frac{x+e^{-i\varphi }}{x+e^{i\varphi }}\right)}^{m}\right]\sin\varphi d\varphi . \end{array}$$

Finally, using Equation (A4), we can easily prove Equation (19) by directly calculating the conjugate.

Finally, we we prove Equation (20). We first rewrite ${\left[K_{m}^{\pm }\left(-x\right)\right]}^{*}$ as

(A5) $${\left[K_{m}^{\pm }\left(-x\right)\right]}^{*}=\int_{0}^{\pi }\left[1-{\left(\frac{x+\left(\pm \right)e^{-i\varphi }}{x+\left(\pm \right)e^{i\varphi }}\right)}^{m}\right]\sin\varphi d\varphi$$

by using Equations (A1) and (A2). By introducing the new variable $\varphi^{\prime }=-\varphi +\pi$, Equation (A5) can be transformed into

(A6) $$\begin{array}{cc} \; & {\left[K_{m}^{\pm }\left(-x\right)\right]}^{*}=\int_{0}^{\pi }\left[1-{\left(\frac{x+\left(\pm \right)e^{-i\left(-\varphi^{\prime }+\pi \right)}}{x+\left(\pm \right)e^{i\left(-\varphi^{\prime }+\pi \right)}}\right)}^{m}\right]\sin\left(-\varphi^{\prime }+\pi \right)d\left(-\varphi^{\prime }+\pi \right)\;\\ \; & =-\int_{\pi }^{0}\left[1-{\left(\frac{x-\left(\pm \right)e^{i\varphi^{\prime }}}{x-\left(\pm \right)e^{-i\varphi^{\prime }}}\right)}^{m}\right]\sin\varphi^{\prime }d\varphi^{\prime }=\int_{0}^{\pi }\left[1-{\left(\frac{x-\left(\pm \right)e^{i\varphi }}{x-\left(\pm \right)e^{-i\varphi }}\right)}^{m}\right]\sin\varphi d\varphi \;\\ \; & =K_{m}^{\pm }\left(x\right). \end{array}$$
